# Multilevel Analysis of Determinants of Anemia Prevalence among Children Aged 6–59 Months in Ethiopia: Classical and Bayesian Approaches

**DOI:** 10.1155/2018/3087354

**Published:** 2018-06-03

**Authors:** Kemal N. Kawo, Zeytu G. Asfaw, Negusse Yohannes

**Affiliations:** ^1^Department of Statistics, Madda Walabu University, Robe, Ethiopia; ^2^School of Mathematical and Statistical Sciences, Hawassa University, Hawassa, Ethiopia; ^3^Department of Statistics, Dilla University, Dilla, Ethiopia

## Abstract

**Background:**

Anemia is a widely spread public health problem and affects individuals at all levels. However, there is a considerable regional variation in its distribution.

**Objective:**

Thus, this study aimed to assess and model the determinants of prevalence of anemia among children aged 6–59 months in Ethiopia.

**Data:**

Cross-sectional data from Ethiopian Demographic and Health Survey was used for the analysis. It was implemented by the Central Statistical Agency from 27 December 2010 through June 2011 and the sampling technique employed was multistage.

**Method:**

The statistical models that suit the hierarchical data such as variance components model, random intercept model, and random coefficients model were used to analyze the data. Likelihood and Bayesian approaches were used to estimate both fixed effects and random effects in multilevel analysis.

**Result:**

This study revealed that the prevalence of anemia among children aged between 6 and 59 months in the country was around 42.8%. The multilevel binary logistic regression analysis was performed to investigate the variation of predictor variables of the prevalence of anemia among children aged between 6 and 59 months. Accordingly, it has been identified that the number of children under five in the household, wealth index, age of children, mothers' current working status, education level, given iron pills, size of child at birth, and source of drinking water have a significant effect on prevalence of anemia. It is found that variances related to the random term were statistically significant implying that there is variation in prevalence of anemia across regions. From the methodological aspect, it was found that random intercept model is better compared to the other two models in fitting the data well. Bayesian analysis gave consistent estimates with the respective multilevel models and additional solutions as posterior distribution of the parameters.

**Conclusion:**

The current study confirmed that prevalence of anemia among children aged 6–59 months in Ethiopia was severe public health problem, where 42.8% of them are anemic. Thus, stakeholders should pay attention to all significant factors mentioned in the analysis of this study but wealth index/improving household income and availability of pure drinking water are the most influential factors that should be improved anyway.

## 1. Introduction

Anemia is a condition characterized by a low level of hemoglobin in the blood [1]. Anemia is a widespread public health problem, and severe anemia is a significant cause of childhood mortality [2]. The World Health Organization (WHO) considers anemia prevalence over 40% as a major public health problem, between 20 and 40% as a medium-level public health problem, and between 5% and 20% as a mild public health problem [1]. High prevalence of anemia and its consequences on children's health, especially for their growth and development, have made anemia an important public health problem, given the difficulty in implementing effective measures for controlling it [3]. Therefore, it is important to understand the scope and strength of individual risk factors for anemia in populations where anemia is common to design more effective interventions [3].

According to [4] above 1.62 billion people were anemic worldwide, and approximately two-thirds of preschool children in Africa and South East Asia were anemic. According to the WHO report, more than half of the world's preschool-age children (56.3%) reside in countries where anemia is a major public health problem [5]. In Sub-Saharan Africa, much of the national prevalence is estimated to be above 40% among this group [6]. In Ethiopia, more than four out of ten children under five (44%) were anemic [7]. From these, about 21% of children were mildly anemic, 20% were moderately anemic, and 3% were severely anemic. Even if the national anemia prevalence estimate has dropped by 19 percent, from 54 percent in 2005 to 44 percent in 2011, it was a major public health problem according to the WHO criteria.

Discussion about model comparison of multilevel model by using classical and Bayesian approaches is rare in literature. However, [8] examined the distribution of weighted anemia prevalence across different groups and performed logistic regression to assess the association of anemia with different factors based on BDHS (2011) data on hemoglobin (Hb) concentration among the children aged 6–59 months. Also [9] conducted a study on the determinants of anemia among children aged 6–59 months living in Kilte Awulaelo Woreda, Northern Ethiopia. Bivariate and multivariate logistic regression analyses were performed to identify factors related to anemia. In this paper we shall consider multilevel analysis of determinants of anemia prevalence among children aged 6–59 months in Ethiopia using classical and Bayesian approaches.

The main concerns of authors were to identify the major determinants and assess the prevalence of anemia among children aged 6–59 months in Ethiopia using classical and Bayesian approaches, that is,

- to identify significant predictors of having high prevalence of anemia among children aged 6–59 months in Ethiopia through classical and Bayesian approach,
- to analyze the within- and between-regions variation of prevalence of anemia among children aged 6–59 months in Ethiopia,
- to make model comparison and suggest an appropriate model for analyzing anemia prevalence in Ethiopia.

## 2. Materials and Methods

### 2.1. Data

This study used the data collected in the Ethiopian Demographic and Health Survey [7]. The Ethiopia Demographic and Health Survey was conducted by the Central Statistical Agency (CSA) under the auspices of the support of the Ministry of Health from 27 December 2010 through June 2011 with a nationally representative sample of nearly 17,817 households. The sampling frame used for the EDHS was the Population and Housing Census conducted by the Central Statistical Authority (CSA) in 2007; during the 2007 Population and Housing Census, each of the kebeles was subdivided into convenient areas called census enumeration areas (EAs). The EDHS sample was selected using a stratified, two-stage cluster design and EAs were the sampling units for the first stage. For the 2011 EDHS, a representative sample of approximately 17,817 households from 624 clusters was selected. In the first stage, 624 clusters, 187 urban and 437 rural, were selected from the list of enumeration areas based on sampling frame. In the second stage, a complete listing of households was carried out in each selected cluster. For this study 5,507 children were included in the analysis after all incomplete observations have been deleted from the data among 9,157 total children held with hemoglobin (Hb) data.

### 2.2. Ethical Approval

Our study was wholly based on an analysis of existing public domain health survey data sets obtained from EDHS 2011, which is freely available online with all identifier information removed. The EDHS 2011 was reviewed and approved by the ICF Macro Institutional Review Board and the National Research Ethics Committee of the Ethiopian Medical Research Council.

### 2.3. Variables in the Study

Variables considered in this study were selected based on literatures which have been conducted at the global level. Potential determinant factors expected to be correlated with anemia status were included as variables of the study.

### 2.4. Response Variable

Hemoglobin is necessary for transporting oxygen to tissues and organs in the body. Hemoglobin analysis was carried out onsite using a battery-operated portable HemoCue analyzer. Parents of children with a hemoglobin level under 11 g/dl were instructed to take the child to a health facility for follow-up care. Unadjusted hemoglobin values are obtained using the HemoCue instrument. Given that hemoglobin requirements differ substantially depending on altitude, an adjustment to sea-level equivalents has been made before classifying children by level of anemia. Prevalence of anemia, based on hemoglobin levels is adjusted for altitude by hemoglobin in grams per decilitre (g/dl) [7]. The response variable of this study was anemia status of children aged 6–59 months in Ethiopia. For the current analysis, response variable (anemia status) was dichotomized indicating whether one is anemic or not.

(1) $$\begin{array}{c} Y_{ij}=\left\{\begin{array}{c} \text{Not anemic}=0\\ \text{Anemic}=1 \end{array}\right\} \end{array}$$

### 2.5. Explanatory Variables

The explanatory variables which might determine the status of anemia of children among 6–59 months were socioeconomic, demographic, health, and environmental factors. From the source of data we considered the following variables region, place of residence, number of children under 5 in the household, wealth index, marital status, child's age in months, sex of children, husband/partner's education level, given iron pills/syrup, source of drinking water, mother's current working status, and child's size at birth.

### 2.6. Common Techniques for Dealing with Missing Data

Missing data is a common problem for almost every health survey data. Missing data presents a problem in statistical analyses. The first issue in dealing with the problem of missing data is determining the missing data mechanism. The work in [10] distinguishes between three types of missing data mechanism; among them we apply missing completely at random (MCAR), which means that missingness is not related to the variables under study. To handle missing value we used listwise deletion which is a common approach and easy to perform by deleting all incomplete observations from the analysis. The results can be unbiased when data are MCAR. Even so, the disadvantage for this method is reduction of sample size.

## 3. Methods of Statistical Analysis

Multilevel models allow the relationship between the explanatory variables at different level and dependent variables at lower level to be estimated, enabling the extent of variation in the outcome of interest to be measured at each level assumed in the model both before and after the inclusion of the explanatory variables in the model.

Two levels of data hierarchy were stated (for instance, individual children of households and regions) in a multilevel logistic regression model. Units at one level are nested within units at the next higher level. In this study the basic data structure of the two-level logistic regression is a collection of N groups (regions) and within group j  (j = 1,2,…N) a random sample *n*_*j*_ of level one units (individual children of households). The response variable is denoted by

(2) $$\begin{array}{c} Y_{ij}=\left\{\begin{array}{cc} 1 & \text{for children having anemia}\\ 0 & \text{for normal}\left(\text{not anemic}\right)\text{children} \end{array}\right. \end{array}$$

with probability *P*_*ij*_ = *P*(*Y*_*ij*_ = 1/*X*_*ij*_, *u*_*j*_) being the probability of children with any anemia for the *i*^*th*^ household in the *j*^*th*^ region and the probability 1 − *P*_*ij*_ = *P*(*Y*_*ij*_ = 0/*X*_*ij*_, ) being the probability of nonanemic (normal) *i*^*th*^ children for the households in the *j*^*th*^ regions. Here, *Y*_*ij*_ follows a Bernoulli distribution.

### 3.1. The Variance Components Model

The variance component two-level model for a dichotomous outcome variable refers to a population of groups (level-two units (regions)) and specifies the probability distribution for group-dependent probabilities *P*_*j*_ in *Y*_*ij*_ = *P*_*j*_ + *ϵ*_*ij*_ without taking further explanatory variables into account. We focus on the model that specifies the transformed probabilities *f*(*P*_*j*_) to have a normal distribution. This is expressed, for a general link function *f*(*P*_*j*_), by the formula

(3) $$\begin{array}{c} \log\left[\;\frac{P_{ij}}{1-P_{ij}}\;\right]=\beta_{0j}+U_{0j}, \end{array}$$

where *β*_0_ is the population average of the transformed probabilities and *U*_0*j*_ the random deviation from this average for group j. Intraclass correlation coefficient (ICC) represents the proportion of the total variance that is attributable to between-group differences and it provides an assessment of whether or not significant between-groups variation exists. Then the intraclass correlation coefficient (ICC) at regions level is given by

(4) $$\begin{array}{c} ICC=\rho =\frac{\sigma_{u}^{2}}{\sigma_{u}^{2}+\sigma_{e}^{2}} \end{array}$$

where *σ*_*u*_^2^ is the between-groups variance which can be estimated by *U*_0*j*_ and *σ*_*e*_^2^ is within-group variance [11].

### 3.2. The Random Intercept Model

The random intercept model is used to model unobserved heterogeneity in the overall response by introducing random effects. In the random intercept model the intercept is the only random effect meaning that the groups differ with respect to the average value of the response variable, but the relation between explanatory and response variables cannot differ between groups. The random intercept model expresses the log odds, i.e., the logit of *P*_*ij*_, as a sum of linear functions of the explanatory variables. That is,

(5) logitPij=log⁡Pij1−Pij=β0j+∑h=1kβhxhij,i=1,2,…nj,  j=1,2,…11

where the intercept term *β*_0*j*_ is assumed to vary randomly and is given by the sum of an average intercept *β*_0_ and group-dependent deviations *U*_0*j*_; that is, *β*_0*j*_ = *β*_0_ + *U*_0*j*_.

As a result we have

(6) $$\begin{array}{c} logit\left(\;P_{ij}\;\right)=\beta_{0}+\overset{k}{\underset{h=1}{\sum }}\beta_{h}x_{hij}+U_{0j} \end{array}$$

Solving for *P*_*ij*_,

(7) $$\begin{array}{c} P_{ij}=\frac{e^{\beta_{0}+\sum_{h=1}^{k}\beta_{h}x_{hij}+U_{0j}}}{1+e^{\beta_{0}+\sum_{h=1}^{k}\beta_{h}x_{hij}+U_{0j}}} \end{array}$$

Equation (6) does not include a level one residual because it is an equation for the probability *P*_*ij*_ rather than for the outcome *Y*_*ij*_, where *β*_0*j*_ + ∑_*h*=1_^*k*^*β*_*h*_*x*_*hij*_ is the fixed part of the model. The remaining *U*_0*j*_ is called the random or the stochastic part of the model. It is assumed that the residual *U*_0*j*_ is mutually independent and normally distributed with mean zero and variance *σ*_*u*_^2^ [12].

### 3.3. The Random Coefficients Model

In the random coefficient model both the intercepts and slopes are allowed to differ across the region. Suppose that there are k level one explanatory variables *X*_1_, *X*_2_,…, *X*_*k*_, and consider the model where all X-variables have varying slopes and random intercept. That is,

(8) logitPij=log⁡Pij1−Pij=β0+∑h=1kβhjxhij+U0j+∑h=1kU1jX1ij

where *β*_0*j*_ = *β*_0_ + *U*_0*j*_ and *β*_*hj*_ = *β*_*h*_ + *U*_*hj*_, h-1,2,…,k.

Here the first part of (8), *β*_0_ + ∑_*h*=1_^*k*^*β*_*hj*_*x*_*hij*_, is called the fixed part of the model and the second part *U*_0*j*_ + ∑_*h*=1_^*k*^*U*_1*j*_*X*_1*ij*_ is called the random part of the model.

### 3.4. Parameter Estimation of Multilevel Model

#### 3.4.1. Likelihood Method

The maximum likelihood (ML) method is a general estimation procedure, which produces estimates for the population parameters that maximize the probability of observing the data that are actually observed. Assuming that the conditional distributions of *Y*_*ij*_ given the random effect *U*_*j*_ are independent of each other, the conditional density of *Y*_*ij*_ is given by *P*_*ij*_:

(9) $$\begin{array}{c} Y_{y_{ij}/u_{j}}=\left(\;\frac{y_{ij}}{u_{ij}}\;\right)\sim Bernoulli \end{array}$$

For two-level logistic Bernoulli response model, where random effects are assumed to be multivariate normal and independent across units, the marginal likelihood function is given by

(10) $$\begin{array}{c} l\left(\;\beta ,\Omega \;\right)=\underset{i}{\prod }f\underset{i}{\prod }\left[\;{\left(\;\pi_{ij}\;\right)}^{y_{ij}}{\left(\;1-\pi_{ij}\;\right)}^{1-y_{ij}}\;\right] \end{array}$$

where *Ω* is variance covariance matrix.

(11) $$\begin{array}{c} \pi_{ij}=\left[\;1+\exp\left(\;-x_{ij}\beta_{j}\;\right)\;\right],\;\beta_{j}=\beta +U_{j} \end{array}$$

*f*(*U*_*ij*_, *Ω*) is typically assumed to be the multivariate normal density and can be written in the form ∫*p*(*U*_*j*_)*f*(*U*_*j*_)*du*_*j*_.

### 3.5. Bayesian Modeling

Bayesian inference involves creating a complete probability model over all data and parameters of interest, fitting the model to observed data, and then reasoning about either the fitted parameters or about new data taking into account the uncertainty in the fitted parameters. In a Bayesian formulation the uncertainty about the value of each parameter can be represented by a probability distribution, if prior knowledge can be quantified [13]. In Bayesian approach, either mean or median of the posterior samples for each parameter of interest is reported as a point estimate. 2.5% and 97.5% percentiles of the posterior samples for each parameter give a 95% posterior credible interval (interval within which the parameter lies with probability 0.95).

### 3.6. The Likelihood Function

The key ingredients to a Bayesian analysis are the likelihood function, which reflects information about the parameters contained in the data, and the prior distribution, which quantifies what is known about the parameters before observing data. The prior distribution and likelihood can be easily combined to form the posterior distribution, which represents total knowledge about the parameters after the data have been observed. Bayesian multilevel logistic analysis specifies a dichotomous dependent variable as a function of a set of explanatory variables. The likelihood contribution from the *i*^*th*^ subject in the *j*^*th*^ group is Bernoulli:

(12) $$\begin{array}{c} Bernoulli\left(\;p_{ij}\;\right)=p_{ij}^{y_{ij}}{\left(\;1-p_{ij}\;\right)}^{1-y_{ij}}, \end{array}$$

where *p*_*ij*_ represents the probability of the event for subject i in j group that has covariate vector *x*_*ij*_ and *y*_*ij*_ indicates the presence (*y*_*ij*_ = 1) or absence (*y*_*ij*_ = 0) of the event for that subject. In multilevel logistic regression, we know that

(13) $$\begin{array}{c} p_{ij}=\frac{e^{\beta_{0}+\beta_{1}x_{1ij}+\beta_{2}x_{2ij}+\cdots +\beta_{k}x_{kij}+U_{0j}}}{1+e^{\beta_{0}+\beta_{1}x_{1ij}+\beta_{2}x_{2ij}+\cdots +\beta_{k}x_{kij}+U_{0j}}} \end{array}$$

where *β*_0_ + *β*_1_*x*_1*ij*_ + *β*_2_*x*_2*ij*_ + ⋯+*β*_*k*_*x*_*kij*_ is fixed part of the model and *U*_0*j*_ is random part of the model and *U*_0*j*_ ~ *N*(0, *σ*_*u*_^2^).

*p*_*ij*_ is the probability of *i*^*th*^ child in *j*^*th*^ group (region) being anemic, so that the likelihood contribution for the *i*^*th*^ subject in the *j*^*th*^ region is

(14) Ly ∣ βi,σu2=eβ0+β1x1ij+β2x2ij+⋯+βkxkij+U0j1+eβ0+β1x1ij+β2x2ij+⋯+βkxkij+U0jyij·1−eβ0+β1x1ij+β2x2ij+⋯+βkxkij+U0j1+eβ0+β1x1ij+β2x2ij+⋯+βkxkij+U0j1−yij

Since individual subjects in the group are assumed to be independent of each other, the likelihood function over a data set of n subjects in the 11 region is then

(15) Ly ∣ βi,σu2=

(16) ∏i=1n∏j=111eβ0+β1x1ij+β2x2ij+⋯+βkxkij+U0j1+eβ0+β1x1ij+β2x2ij+⋯+βkxkij+U0jyij·1−eβ0+β1x1ij+β2x2ij+⋯+βkxkij+U0j1+eβ0+β1x1ij+β2x2ij+⋯+βkxkij+U0j1−yij

### 3.7. Prior Distribution

The prior distribution is a probability distribution that represents the prior information associated with the parameters of interest. There are two types of prior distribution: informative priors and noninformative priors.

### 3.8. Model Comparison

In this study Akaike information criterion (AIC) and Bayesian information criterion (BIC) were used for model comparison. A model with a lower AIC and BIC is preferred over a model with a larger AIC and BIC.

### 3.9. Software Used

The statistical software types used in this study were SPSS version 20 (StataCorp, Texas 77845, USA) and WinBUGS14. SPSS was used for the descriptive analysis, STATA was used for multilevel analysis part, and WinBUGS14 was used for Bayesian analysis.

## 4. Results and Discussions

The results of the analysis are divided into descriptive analysis and multilevel binary logistic models from categorical data. Results and their discussions are presented in the following sections.

### 4.1. Descriptive Analysis

Descriptive statistics are a set of brief descriptive figures that summarizes a given data set, which can be a representation of entire sample. A total sample of 5,507 children aged between 6 and 59 months was included in this study. Among these, 2358 (42.8%) were anemic (Hb < 11.0 g/dl) while 3149 (57.2%) were not anemic at the date of the survey.

### 4.2. Bivariate Analysis between Response and Predictors

This section reports the association between the response variable and each predictor variable. The bivariate analysis, based on Pearson's chi-square statistic, provides a preliminary insight into the association/relationship between all selected independent variables and dependent variable. High values of Pearson chi-square for a given independent variable indicate that there is strong association between each of the given independent variables and the dependent variable keeping the effect of the other factors constant. That is, testing the hypotheses are as follows:

 
*H*_*o*_ = there is no association between the dependent and independent variables.
 
*H*_1_ = there is association between the dependent and the particular independent variable.

The decision was based on the chi-square value and p value at 0.05 level of significance.

Basic descriptive information that summarizes the association between predictors and response variable is presented in Table 1. The results in Table 1 show the row percentage and count of anemic/not anemic status of children aged 6–59 months with respect to the categorical covariates.

The bivariate association between anemia status of children aged 6–59 months and predictors shown in Table 1 indicates that anemia status was strongly associated with region, place of residence, number of children under 5 in the household, wealth index, mother's marital status, child's age in months, husband/partner's education level, given iron pills/syrup, source of drinking water, mother's current working status, and child's size at birth. Sex of the children was the only explanatory variable that had no significant association with anemia status among children aged 6–59 months. The prevalence of anemia among children aged between 6 and 59 months varied from one region to another in Ethiopia. The highest proportion of anemia status among children aged 6–59 months was observed in Afar (67.2%) followed by Somali (61.8%) and Dire Dawa (50.5%) as opposed to the lowest percentage that was recorded in Addis Ababa (28.4%) followed by SNNP (35.1%) and Tigray (35.8). The regional variation of anemia prevalence among children ranges from low (Addis Ababa) to high (Afar).

This regional variation is very realistic and expected based on living conditions of people within and between regions. People who are living in Afar and Somalia are nomadic and the main daily meal of thus people is milk. A number of previous studies documented that the effect of milk can be a cause of anemia.

The prevalence of anemia among children aged 6–59 months also differs by type of place of residence. Accordingly, higher numbers of anemic children (44.4%) resided in rural areas, and relatively small number of anemic children (38.3%t) resided in urban areas. This is quite consistent with other similar studies conducted in Ghana, where prevalence of anemia among rural children is 17% higher than that of urban children [13]. Moreover, there is a significant variation within and between the continents. Even prevalence of anemia reported from several developing countries varied. It is about 16.1% in Philippines and 87% in Tanzania [14, 15]. The result presented in Table 1 also reveals that the prevalence of anemia among children aged between 6 and 59 months varied by families educational level. The highest percentage of anemia was observed in children whose families have no education (48.1%) as opposed to the lowest percentage of anemia which was recorded for children whose families have secondary education and above (28%) followed by primary education level (39.4%). It indicates that children of more educated families were less exposed to anemia compared to children of uneducated families.

The total number of children under five in the household is also another important variable. The prevalence of anemia among children aged between 6 and 59 months was 36.5% for children under five with total number of 0-3 in the household, 45.8% for children under five with total number of 4-6 in the household, and 46.9% for children under five with total number above 6 in the household. Hence, as the number of children under five in the household increases the prevalence of anemia among children aged between 6 and 59 months in Ethiopia was increased. The wealth index is a composite measure of the cumulative living standard of a household used as a proxy for socioeconomic status. It was calculated using household's ownership of selected assets by CSA. The result in Table 1 indicates that wealth index was found to have a significant association with anemia status among children aged between 6 and 59 months at 5% level of significance and 49.5%, 40.9%, and 35.6% of children aged between 6 and 59 months from poor, middle, and rich households were anemic, respectively. It indicates that children from rich families were less exposed to anemia compared to children from poor families. With regard to marital status, the higher percentage of anemia among children aged between 6 and 59 months was observed among children whose families were married (43.5%) followed by children whose families were widowed (40.7%) and lower proportion of anemia was observed for children whose families were divorced/separated (35.5%).

The proportion of anemia among children aged between 6 and 59 months, observed in Table 1, also differs with their age groups. For instance, higher proportion of anemia was observed for children under 6–23 months of age (50.5%) and the lowest proportion of anemia was found in the age group between 42 and 59 months (36.7%) followed by children under 24–41 months (42.7%). This finding is similar to study in Brazil, Bangladesh, and Northern Ethiopia [9, 16, 17]. Hence, as age increased the prevalence of anemia among children aged between 6 and 59 months in Ethiopia was decreased. Given iron pills/syrup is another important variable that was strongly associated with anemia status among children aged between 6 and 59 months. The proportion of anemia status was also high for children who did not receive iron pills/syrup. Children, who received iron pills/syrup, had lower risk of anemia (36.1%) than children who did not receive iron pills/syrup (44.4%). The result in Table 1  indicates that size of child at birth was found to have significant association with anemia status among children aged between 6 and 59 months at 5% level of significance and 48.0%, 39.2%, and 42,4% of children with size larger than average, equal to average, smaller than average were anemic, respectively. This indicates the prevalence of anemia among children aged between 6 and 59 months was higher for children with size smaller than average and larger than average compared to children with average size at birth.

From result presented in Table 1, source of drinking water had significant association with anemia status among children aged between 6 and 59 months in Ethiopia. The proportion of anemia status was also high for children who did not use improved water. Children, who used improved water, had lower risk of anemia (38.1%) than children who did not use improved water (44.3%). This finding supported by the study conducted in the Philippines shows association between anemia and water supply [14]. Mother's current working status also had significant association with anemia status among children aged between 6 and 59 months in Ethiopia.

The main problem with the bivariate approach is that it ignores the possibility that a collection of variables, each of which could be weakly associated with the outcome, can become an important predictor of the outcome when taken together [18]. Hence, logistic regression approach that takes into account the drawback mentioned by the bivariate technique is considered in this analysis.

Thus, authors considered multilevel logistic regression in both approaches, classical and Bayesian perspectives.

### 4.3. Results of Multilevel Models

Before directly going to the analysis of multilevel models the first important step is variable selection. For this study we have used stepwise variable selection technique to determine the variables to be included in the multilevel models. Accordingly, of the total variables considered, twelve variables (region, place of residence, number of children under 5 in the household, wealth index, marital status, child's age in months, sex of children, husband/partner's education level, given iron pills/syrup, source of drinking water, mother's current working status, and child's size at birth) are selected to be included in the multilevel model.

#### 4.3.1. Variance Components Model

We first fitted a simple model with no predictors, i.e., variance components model that predicts the probability of anemia status.

The variance components model results in Table 2 revealed the information of the fixed effect; authors can say that the estimated average log odds of anemia status among children aged between 6 and 59 months across regions of the country are *β*_0_ = −0.2849. Authors can convert this back to a probability 0.428; this is the overall proportion of prevalence of anemia among children aged between 6 and 59 months in Ethiopia without accounting for other sources of variation. The intercept for region j is −0.2849 + *U*_0*j*_, where the variance of *U*_0_ between regions in region average log odds of being anemic is estimated as ${\hat{\sigma }}_{u}^{2}=0.2126$.

We reject null hypothesis which states that the variation across region was zero. So this result suggests that the variation of prevalence of anemia among children aged between 6 and 59 months due regional difference was nonzero. Hence, we conclude that the regional difference contributed to the variation in prevalence of anemia among children aged between 6 and 59 months in Ethiopia. The variances (*π*^2^/3) (3.29) and 0.2126 estimate the variation among individual children in the household and regions of the country. In variance components model it is possible to decompose variance into regional level (higher level) and individual level. Individual (level 1) variance was to assess how many variations are due to individuals themselves and how many variations are due to regional level. According to [19] the individual (level 1) variance was fixed to (*π*^2^/3) (3.29) for logit model.

In order to get an idea of how many variations in prevalence of anemia among children aged between 6 and 59 months were attributable to the region level factors, it is useful to see the intraregional correlation coefficient. The intraregional correlation coefficient (ICC) in variance components model is ICC = 0.0607, meaning that roughly 6.07% of the total variability in prevalence of anemia among children aged between 6 and 59 months is significantly attributable to the regional level, whereas the remaining 93.03% is attributable to individual level (i.e., within-region differences).

#### 4.3.2. Random Intercept Model

To identify the effect of explanatory variables a multilevel binary logistic model with random intercept and fixed explanatory variables was estimated. The deviance-based chi-square for significance overall goodness fit model (*χ*^2^ = −2log (likelihood of variance component model)-(−2log (likelihood of random intercept model)) is 224.11, P < 0.05) indicates that the random intercept model with the fixed explanatory variables is found to be a better fit as compared to the variance component model discussed in the previous section. Note that there is change (decrease) in the estimate of the between-region variance from variance component model 0.2126 to random intercept model 0.1698, suggesting that the distribution of fixed explanatory variables is somewhat different across regions of the country.

The results from the random intercept model in Table 3 showed that the random intercept (*β*_0_) is significant implying that the average proportion of anemia among children aged between 6 and 59 months differs from region to region. The intercept estimation is random at the regional level, var(*U*_0*j*_). Thus, the value of var(*U*_0*j*_) = *σ*_*u*_^2^ = 0.1698 is the estimated variance of the intercept. The multilevel logistic regression analysis result displayed in Table 3 confirmed the significance of regional difference in prevalence of anemia among children aged between 6 and 59 months in Ethiopia. The deviance-based chi-square = 224.11, p value < 0.001 for random effects in random intercept model, suggesting that children with the same characteristics in different regions have different anemia status in Ethiopia; that is, there is a clear regional effect.

The results displayed in Table 3 showed that the intraregional correlation coefficient (ICC) is estimated as $\hat{\rho }$ = 0.0491, meaning that roughly 4.91% of the total variability in prevalence of anemia among children aged between 6 and 59 months is attributable to the regional level, with the remaining unexplained 95.09% being due to individual differences. From Table 3, the analysis of multilevel binary logistic regression revealed that the prevalence of anemia among children aged between 6 and 59 months varied among regions. In addition, the number of children under five in the household, wealth index, child's age in month, mother's current working status, husband/partner's education level, given iron pills/syrup, size of child at birth, and source of drinking water were also found to be significant determinants of variation in prevalence of anemia among children aged between 6 and 59 months, whereas sex of children, place of residence, and marital status were insignificant predictors of variation in prevalence of anemia among children aged between 6 and 59 months. From the random part variance component of the random intercept model *σ*_*u*_^2^ was found to be significant, which implies that regions difference contributes to the variation of prevalence of anemia among children from the random intercept and fixed explanatory model. Accordingly, the test statistics explored that marital status and sex of the children are the only explanatory variables that are found to be insignificant (p > 0.05) factors of the dependent variables (anemia status) among the considered explanatory variables. There was no statistically significant difference of the sex in terms of prevalence. This result maintains what is stated with studies done in [19, 20].

The prevalence of anemia among children aged between 6 and 59 months, those from middle and rich families, decreased by 17% and 28%, respectively, as compared to those from poor families (ref) controlling for other variables in the model. This could be because families with middle and rich economic level can afford basic requirements such as clear water, medical care, and sufficient food/nutritional requirements needed for proper children health compared to those families with poor economic level who are unable to provide the basic needs. This finding is consistent with a previous study conducted in Bangladesh [8].

This study also revealed that the values 0.2645 and 0.1735 are increasing in log odds of anemia status among children aged 6–59 months in the household with total number of children under five being 4-6 and above six, respectively; the odds ratio being exp⁡(0.2645) = 1.30 and exp⁡(0.1735) = 1.19, respectively, means that odds of anemic children in the household with total number of children being 4-6 and above six were 1.30 times and 1.19 times higher compared to children in the household in group with total number of 0-3 (ref) keeping other variables in the model constant. It is implied that the prevalence of anemia among children aged between 6–59 months in Ethiopia was higher in the households with more number of children under five relative to households with lower number of children under five. This result maintains what is stated by studies done by [21].

The odds of prevalence of anemia among children aged between 6 and 59 months whose families have primary and secondary/above level of education reduced by 13.78% and 50.62%, respectively, as compared to children of uneducated family (ref) controlling for other variables in the model. This implies that families education is an important socioeconomic characteristic of anemia status; that is, the prevalence of anemia among children aged between 6 and 59 months in the country decreases as family's education level increases, because educated families give attention to their status. They also provide clear environment to children, are more likely to have more knowledge about care taking, and have higher demands. This finding is consistent with the previous studies done in Ethiopia [22].

Likewise, the odds of anemia status among children aged between 6 and 59 months of children who had been given iron pills/syrup were significantly different from children who had not been given iron pills/syrup (ref). A child who had been given iron pills/syrup was 0.7324 times less [OR: 0.7324; 95% CI: 0.6352, 0.8674] likely to have anemia compared with children who had not been given iron pills/syrup (ref). Or from the multilevel binary logistic regression analysis the value −0.2981 ($\hat{\beta }$) decreases log odds of anemia status among children aged between 6 and 59 months who had been given iron pills/syrup [OR: 0.7324, 95% CI: (0.6352, 0.8674)]. It means that odds of the prevalence of anemia among children aged between 6 and 59 months had statistically decreased by 26.76% for children who had been given iron pills/syrup. Iron deficiency is the main factor for the prevalence of anemia. This finding is consistent with the previous studies done by [23].

The current study found that the prevalence of anemia among children aged between 6 and 59 months is significantly associated with age of children. For a one-step increase in age category (to age group 24-41), the log odds of prevalence of anemia in children aged 6–59 months were decreased by 26.73% when compared with age group of 6–23 (ref). For a one-step increase in age category (to 42-59), the log odds of prevalence of anemia in children aged 6–59 months were decreased by 44.54% compared with age group of 6–23 (ref). Therefore, children are more likely to be anemic in age category of 6–23 as compared to other age categories. This indicates that the prevalence of anemia among children aged between 6 and 59 months in Ethiopia is more likely in their early ages of 6–23 (ref). This result maintains what is stated with studies done by [21, 24].

#### 4.3.3. Results of Random Coefficients Model

It is possible to generalize the model so that the effect of level 1 covariates is different in each region. This can be done by adding random coefficients in front of some of the individual-level covariates of the model. This model contains a random slope for source of drinking water and family education level, which means that it allows the effect of the coefficient of the explanatory variable to vary from region to region.

By adding level 1 predictors, the ICC increased and is estimated as $\hat{\rho }$ = 0.075, meaning that roughly 7.5% of the total variability in prevalence of anemia among children aged between 6 and 59 months is attributable to the random factor and region in random coefficient multilevel binary logistic model. From Table 4 the random coefficient estimates for intercepts and the slopes vary significantly at 5% significance level, which implies that there is a considerable variation in the effects of family education level and source of drinking water; these variables differ significantly across the regions. The variance of intercept in the random slope model is 0.2303, which is still large, relative to its standard error of 0.1070. Thus there remains some regional level variance unaccounted for in the model. The variance corresponding to the slope of source of drinking water is 0.0066, which is relatively small with respect to its standard error; this suggests that the effect of source of drinking water may be justified in constraining the effect to be fixed. The effect of improved source of drinking water as log odds of prevalence of anemia in region j is estimated as −0.2776 + ${\hat{U}}_{1j}$, and the between-regions variance in the effect of source of drinking water is estimated as 0.0066. Likewise, the variance corresponding to the slope of family education level is 0.0282, which is relatively large with respect to its standard error (SE = 0.0228); thus, this suggests that the effect of family education may be justified in constructing the effect to be random. The effect of secondary education and above as log odds of prevalence of anemia in region j is estimated as −0.7413 + ${\hat{U}}_{1j}$, and the between-regions variance in the effect of secondary education/above was estimated as 0.0282.

The significance of this difference further indicates that a model with a random coefficient is more appropriate to explain regional variation than a model with fixed coefficients. The correlation between the intercept and random slope of source of drinking water is −0.0267. This implies that the prevalence of anemia among children aged between 6 and 59 months who have used improved water was less than among those who have not used improved water by a larger factor at regions with higher intercepts compared to regions with lower intercepts.

#### 4.3.4. Model Comparison

The choice of relevant multilevel model is an important step, and it should be based on the necessity of parsimony in the model. Parsimony means that models should be as simple as possible [25].

As shown in Table 5 the deviance-based chi-square value (*χ*^2^ = 220.49, p value < 0.001) is significant for the variance components model. The deviance-based chi-square value (*χ*^2^ = 224.11, p value < 0.001) is significant for random intercept model which implies that the random intercept model fits better as compared to variance components model. Also the deviance-based chi-square value for random effects (*χ*^2^ = 187.5, p value < 0.001) for multilevel random slope model (random coefficient model) is also statistically significant. Both models seem to be better for the data compared to variance component model. However, based on deviance, AIC and BIC, authors can see that model fit statistic values (AIC = 7098.61 and BIC = 7224.27) for random intercept model were the smallest among models considered. Therefore, the random intercept model better fits the data to predict prevalence of anemia among children aged between 6 and 59 months in Ethiopia.

Comparison between the two prominent statistical thoughts, classical and Bayesian approaches, and then sound conclusion should be made on the best one for having realistic implementation.

### 4.4. Bayesian Multilevel Analysis

The Gibbs sampler algorithm was implemented with 30,000 iterations in three different chains, 10,000 burn-in terms discarded, to obtain 60,003 samples from the full posterior distribution for the multilevel model. We used noninformative normal prior distribution with mean = 0 and precision = 0.001 for the fixed effect and inverse gamma distribution for sigma with *scale* = 0.1, *shape* = 0.1 for the random effect. This implies that the parameters of the covariates were estimated by 60,003 Markov chain sample values, simply using the Markov chain samples after the burn-in state. Assessment of model convergence also was checked through time series plot, kernel density, and Gelman-Rubin statistics. All were confirmed as the model successfully converged.

#### 4.4.1. Results of Bayesian Analysis

The Bayesian parameter estimation method is explored for the selected model and the random intercept model. The results are displayed in Table 6.

From Table 6, the sample obtained from posterior distribution, summary statistics of all parameters for posterior distribution are presented and the predictor variables like age of children, husband/partners' education level, wealth index, number of children under five in the household, mothers' current working status, size of child at birth, given iron pills/syrup, place of residence, and source of drinking water were also found to be significant determinants of anemia among children aged between 6 and 59 months at 5% level of significance (since the credible intervals of these variables do not contain zero (at least for one category)).

The Bayesian multilevel logistic regression analysis result displayed in Table 5 also estimates the random effect at the regional level, var(*u*_0*j*_). Thus, the value of var(*u*_0*j*_) = *σ*_*u*_^2^ = 0.2658 and since 95% credible confident interval does not contain zero it is significant at 5%. This confirmed the significance of regional difference in prevalence of anemia. That is, there is a clear regional effect. The fixed effect parametric multilevel random intercept models in classical approach and in Bayesian approach were fitted. Both methods give almost consistent results, but most of the parameters in Bayesian analysis had smaller standard error than the corresponding classical multilevel random intercept model. Therefore, Bayesian multilevel random intercept model gives better fit than the classical multilevel random intercept models. In the estimation of random effects, there is a difference between the estimation of likelihood and Bayesian approaches, that is, 0.1698 and 0.2658, respectively, and the Bayesian approach estimates more than the likelihood approach in explaining the variation of prevalence of anemia across the region of the country. Bayesian analysis gave consistent estimates with the respective multilevel models and additional solutions as posterior distribution of the parameters convergence.

The results displayed in Table 6 also showed that the intraregional correlation coefficient (ICC) is estimated as $\hat{\rho }=0.074$. This means that about 7.4% of the total variability in prevalence of anemia is due to differences across regions, with the remaining unexplained 92.6% attributable to individual differences.

## 5. Conclusions

The current study confirmed that prevalence of anemia among children aged 6–59 months in Ethiopia was severe public health problem, where 42.8% of them anemic and based on WHO criteria greater than 40% are categorized under severe public health problem. Thus, stakeholders should pay attention to all significant factors mentioned in the analysis of this study but wealth index/improving household income and availability of pure drinking water are the most influential factors that should be improved anyway. Regional variation is the sole finding of this paper and hence potential stakeholders have to give special consideration for children who are living in the highest anemic prevalence regions. Moreover, those households who are living in nomadic region like Afar and Somalia should be trained on the cause of anemia and its consequences.

From the methodological aspect, it was found that multilevel random intercept model is better compared to variance components model and random coefficients model in fitting the data. Bayesian analysis gave consistent estimates with the respective multilevel models and additional solutions as posterior distribution of the parameters.

## Figures and Tables

**Table 1:** Cross tabulation of anemia status versus predictor variables.

Variables	Anemia status				Total	DF	Chi-square	P-value
	Anemic		Not anemic					
	N	%	N	%				
**Region (RGN)**					5507			

Addis Ababa	55	28.4%	139	71.6%	194	10	267.04	0.001
Afar	357	67.2%	174	32.8%	531			
Amhara	244	37.2%	412	62.8%	656			
Oromiya	354	40.5%	519	59.5%	873			
Somali	261	61.8%	161	38.2%	422			
Benishangul-Gumuz	181	37.8%	298	62.2%	479			
SNNPR	279	35.1%	516	64.9%	795			
Gambela	132	37.5%	220	62.5%	352			
Harari	117	41.8%	163	58.2%	280			
Dire Dawa	162	50.5%	159	49.5	321			
Tigray	216	35.8%	388	64.2%	604			

**Place of Residence (POR**					5507			

Urban	534	38.3%	862	61.7%	1396	1	15.92	0.001
Rural	1824	44.4%	2287	55.6%	4111			

**Sex of child (SEX)**					5507			

Male	1237	44.5%	1608	56.5%	2845	1	1.05	0.318
Female	1121	42.1%	1541	57.9%	2662			

**No of children u5 in the household (NOCH)**					5507			

0-3	682	36.5%	1187	63.5%	1869	2	46.62	0.001
4-6	1258	45.8%	1489	54.2%	2747			
above 6	418	46.9%	473	53.1%	891			

**Wealth index (WI)**					5507			

Poor	1078	49.5%	1099	50.5%	2177	2	75.53	0.001
Middle	719	40.9%	1037	59.1%	1756			
Rich	561	35.6%	1013	64.6%	1574			

**Marital status (MS)**					5507			

Married	2100	43.5%	2724	56.5%	4824	2	9.93	0.001
Widowed	122	40.7%	178	59.3	300			
Divorced/separated	136	35.5%	247	64.5	383			

**Child's age in month (AGE)**					5507			

6-23	794	50.5%	779	49.5%	1573	2	67.46	0.001
24-41	859	42.7%	1152	57.3%	2011			
42-59	705	36.7%	1218	63.3%	1923			

**Mother's working status (MWS)**					5507			

Not working	1559	44.4%	1953	55.6%	3512	1	9.61	0.002
Working	799	40.1%	1196	59.9%	1995			

**Education level (PEL)**					5507			

No education	1404	48.1%	1517	51.9%	2921	2	93.18	0.001
Primary	795	39.4%	1224	60.6%	2019			
Secondary & above	159	28.0%	408	72.0%	567			

**Given iron pills (GIP)**					5507			

No	1980	44.4%	2479	55.6%	4459	1	24.08	0.001
Yes	378	36.1%	670	63.9%	1048			

**Size of child at birth (SIZE)**					5507			

Smaller than average	684	42.4%	929	57.6%	1613	2	30.63	0.001
Average	863	39.2%	1341	60.8%	2204			
Larger than average	811	48.0%	879	52.0%	1690			

**Source of drinking water (SDW)**					5507			

Not improved	1861	44.3%	2339	55.7%	4200	1	15.92	0.001
Improved	498	38.1%	809	61.9%	1307			

**Table 2:** Estimates for variance components model (EDHS, 2011).

Fixed effects	Estimate	S. error	z value	p-value
*β*_0_ = intercept	−0.2849	0.1425	−2	0.046^*∗*^

Random effect				

${\hat{\sigma }}_{u}^{2}$	0.2126	0.0477	4.457	0.001

ICC(*ρ*)	0.0608			

**Table 3:** Estimates of random intercept model (EDHS, 2011).

**Fixed effects**	Categories	coefficients	SE	z-value	p-value	$\exp\left(\hat{\beta }\right)$	95% C.I for $\exp\left(\hat{\beta }\right)$	
							lower	upper
*β*_0_-intercept		0.3536	0.1733	2.04	0.041		0.0139	0.6933

Place of Residence	Urban (ref)							
	Rural	0.0773	0.07535	1.03	0.305	1.0804	0.9320	1.252

Sex of child	Male (ref)							
	Female	−0.0355	0.05755	−0.62	0.537	0.9651	0.8622	1.080

Number of children under 5 in household	0-3 (ref)							
	4-6	0.2624	0.066	3.99	0.001	1.3	1.1427	1.479
	above 6	0.1718	0.089	1.92	0.054	1.187	0.9968	1.415

Wealth index	Poor (ref)							
	Middle	−0.1876	0.0699	−2.68	0.007	0.829	0.7228	0.9508
	Rich	−0.3296	0.0788	−4.18	0.001	0.7192	0.6163	0.8393

Marital status	Married (ref)							
	Widowed	0.0654	0.1276	0.51	0.608	1.0676	0.8314	1.371
	Divorced/Separated	−0.0624	0.1177	−0.53	0.596	0.9395	0.7460	1.1833

Child's age in month	6-23 (ref)							
	24-41	−0.3111	0.0714	−4.36	0.001	0.7326	0.6370	0.8426
	42-59	−0.5876	0.0728	−8.08	0.001	0.5556	0.4818	0.6408

Mother's current working status	Not working (ref)							
	Working	−0.1540	.0615	−2.50	0.012	0.8572	0.7599	0.9671

Husband/partner's education level	No education (ref)							
	Primary	−0.1493	0.0646	−2.31	0.021	0.8613	0.7588	0.9776
	Secondary & above	−0.7062	0.1129	−6.25	0.001	0.4935	0.3955	0.6158

Given iron pills/syrup	No (ref)							
	Yes	−0.2993	0.0795	−3.76	0.001	0.7414	0.6344	0.8664

Size of child at birth	Larger than average (ref)							
	Average	−0.1530	0.0701	−2.18	0.029	0.8581	0.7480	0.9845
	Smaller than average	0.1123	0.0747	1.50	0.132	1.1189	0.9666	1.2952

source of drinking water	Not improved (ref)							
	Improved	−0.2685	0.0679	−3.96	0.001	0.7645	0.6693	0.8733

**Random effect**								

${\hat{\sigma }}_{u_{{\;}_{\;}}}^{2}=var(u_{0j})$		0.1698	0.0768	2.21			0.2645	0.6420

ICC(*ρ*)		0.0491						

**Table 4:** Estimates of random coefficients model (EDHS, 2011).

**Fixed effects**	Categories	coefficients	SE	z-value	p-value	$\exp\left(\hat{\beta }\right)$	95% C.I for $\exp\left(\hat{\beta }\right)$	
							lower	upper
Wealth index	Poor (ref)							
	Middle	−0.18087	0.07025	−2.58	0.010	0.8346	0.7273	0.9577
	Rich	−0.3187	0.0792	−4.02	0.001	0.7271	0.6225	0.8492

Number of children under 5	0-3 (ref)							
	4-6	0.2620	0.0659	3.97	0.001	1.2995	1.1420	1.4788
	above 6	0.1656	0.0896	1.85	0.065	1.1800	0.9899	1.4067

Place of Residence	Urban (ref)							
	Rural	0.0732	0.0757	0.97	0.334	1.0759	0.9276	1.2480

Marital status	Married (ref)							
	Widowed	0.0816	0.1281	0.64	0.524	1.0850	0.8441	1.3950
	Divorced/Separated	−0.0619	0.11767	−0.526	0.599	0.9410	0.7470	1.1855

Child's age in month	6-23 (ref)							
	24-41	−0.3061	0.0715	−4.28	0.001	0.7363	0.6401	0.8471
	42-59	−0.5852	0.0729	−8.03	0.001	0.5570	0.4829	0.6425

Mother's current working status	Not working (ref)							
	Working	−0.1542	0.0616	−2.50	0.012	0.8571	0.7596	0.9671

Husband/partner's education level	No education (ref)							
	Primary	−0.1578	0.0844	−1.87	0.061	0.8540	0.7238	1.0076
	Secondary & above	−0.7413	0.1548	−4.79	0.001	0.4765	0.3518	0.6454

Given iron pills/syrup	No (ref)							
	Yes	−0.2943	0.0796	−3.70	0.001	0.7450	0.6374	0.8708

Size of child at birth	Larger than average (ref)							
	Average	−0.1561	0.0702	−2.22	0.026	0.8555	0.7455	0.9816
	Smaller than average	0.1044	0.0744	1.40	0.161	1.1100	0.9594	1.2844

source of drinking water	Not improved (ref)							
	Improved	−.2776	.0736	−3.77	0.001	0.7576	0.6558	0.8752

*β*_0_-intercept		0.3431	0.1651	2.08	0.038		0.0196	0.667

**Random effect**								

*σ*_0_ _ ___^2^ = var(*U*_0*j*_)	0.2303	0.1070					0.0927	0.573

*σ*_1_ _ ___^2^ = var(*U*_1*j*_)	0.0282	0.0228					0.0058	0.1380

*σ*_2_ _ ___^2^ = var(*U*_2*j*_)	0.0066	0.027					2.10e − 06	21.043

*σ*_12_ _ ___ = cov(*U*_1*j*_, *U*_2*j*_)	0.0075	0.0192					−0.0302	0.0452

*σ*_10_ _ ___ = cov(*U*_1*j*_, *U*_0*j*_)	−0.0571	0.0415					−0.1384	0.0242

*σ*_20_ _ ___ = cov(*U*_2*j*_, *U*_0*j*_)	−0.0267	0.0397					−0.1046	0.0512

ICC(*ρ*)	0.075							

**Table 5:** Model comparison.

Fitted model	**Variance component model**	**Random intercept model**	**Random coefficients model**
−log likelihood	−3650.20	−3530.50	−3527.18

Deviance-based chi-square	220.49	224.11	187.5

P-value	0.001	0.001	0.001

AIC	7304.4	7098.61	7100.36

BIC	7317.63	7224.27	7252.48

**Table 6:** Bayesian estimates for random intercept model.

**Fixed part**	**categories (level)**	**node**	**mean**	**Sd**	**MC error**	**2.5%**	**median**	**97.5%**
Intercept		alpha	0.3487	0.1735	0.006318	0.1714	0.349	0.5245

Sex of child	Male (ref)							
	Female	beta[1]	−0.03455	0.05672	3.674E − 4	−0.1483	−0.03459	0.07822

Child's age in month	6-23 (ref)							
	24-41	beta[2]	−0.2962	0.07136	6.348E − 4	−0.4352	−0.2965	−0.1557
	42-59	beta[3]	−0.5756	0.07284	6.278E − 4	−0.7187	−0.5759	−0.4328

Place of Residence	Urban (ref)							
	Rural	beta[4]	0.1743	0.07371	9.129E − 4	0.03031	0.1742	0.3196

No of children u5	0-3 (ref)							
	4-6	beta[5]	0.2947	0.06551	6.801E − 4	0.158	0.2946	0.4308
	above 6	beta[6]	0.2098	0.08454	7.711E − 4	0.02496	0.2096	0.3946

Wealth index	Poor (ref)							
	Middle	beta[7]	−0.218	0.06826	5.559E − 4	−0.355	−0.2182	−0.08018
	Rich	beta[8]	−0.4143	0.07668	7.599E − 4	−0.566	−0.4138	−0.2648

Marital status	Married (ref)							
	Widowed	beta[9]	0.04532	0.128	5.975E − 4	−0.2066	0.04626	0.2932
	Divorced/Separated	beta[10]	−0.09123	0.1178	5.506E − 4	−0.3231	−0.0906	0.1392

Education level	No education (ref)							
	Primary	beta[11]	−0.07366	0.08864	0.001167	−0.09975	−0.07367	−0.03468
	Secondary	beta[12]	−0.1211	0.08781	0.001177	−0.05145	−0.1212	−0.02918

Mother's current working status	Not working (ref)							
	Working	beta[13]	−0.2006	0.07009	7.051E − 4	−0.3391	−0.2001	−0.06329

Given iron pills/syrup	No (ref)							
	Yes	beta[14]	−0.2927	0.07935	4.086E − 4	−0.4492	−0.2926	−0.138

Size of child at birth	Larger than average (ref. cat)							
	Average	beta[15]	−0.1473	0.07012	6.419E − 4	−0.2848	−0.1474	−0.01014
	Smaller than average	beta[16]	0.1308	0.07479	6.401E − 4	−0.01628	0.1311	0.2775

source of drinking water	Not improved (ref)							
	Improved	beta[17]	−0.2641	0.06788	3.453E − 4	−0.3978	−0.2639	−0.1319

**Random effects**								

sigma		*σ*_*u*_^2^	0.2658	0.1566	0.0016	0.09924	0.2266	0.6693

## Data Availability

The analysis in this study is based on data available from the Ethiopian Demographic and Health Survey.
